# Buttock Necrosis after Uterine Artery Embolization for Delayed Hysterectomy in Placenta Percreta

**DOI:** 10.1155/2016/6921280

**Published:** 2016-12-06

**Authors:** Devin D. Smith, Annette Perez-Delboy, William M. Burke, Ana I. Tergas

**Affiliations:** ^1^Department of Obstetrics and Gynecology, Columbia University College of Physicians and Surgeons, New York, NY, USA; ^2^Herbert Irving Comprehensive Cancer Center, Columbia University College of Physicians and Surgeons, New York, NY, USA; ^3^Department of Epidemiology, Mailman School of Public Health, Columbia University, New York, NY, USA

## Abstract

*Background*. Morbidly adherent placenta (MAP) is increasing in incidence and is commonly associated with maternal hemorrhage and cesarean hysterectomy. Uterine artery embolization (UAE) may be utilized in the conservative management of placenta percreta to potentially reduce blood loss. The incidence of complications from UAE in the conservative management of placenta percreta is poorly described. To our knowledge, we present the first reported case of buttock necrosis in this setting.* Case*. A 39-year-old gravida nine para two with placenta percreta who underwent conservative management with UAE complicated by right buttock necrosis.* Conclusion*. While UAE may potentially decrease blood loss, it is not without risk. More studies must be performed in order to quantify those risks and determine the clinical utility of UAE.

## 1. Introduction

Morbidly adherent placenta (MAP) is a spectrum of disorders caused by the abnormally deep invasion of placental tissue into the uterine myometrium. It includes placenta accreta, increta, and percreta and is thought to be due to myometrial scarring and abnormal vascularization following uterine surgery, which leads to excessive trophoblastic invasion [1]. While historically it is a rare diagnosis, MAP is now one of the leading causes of obstetric maternal morbidity. Recent studies have reported an incidence as high as 1 in 533 pregnancies [1]. MAP is commonly associated with catastrophic maternal hemorrhage and cesarean hysterectomy. The incidence of maternal mortality associated with MAP has been reported to be as high as 7% [2].

Successful management of MAP hinges upon timely antenatal diagnosis and planning, as well as optimization of surgical management. In the management of placenta percreta, operative morbidity may be decreased by leaving the placenta in situ [1]. Delayed hysterectomy is thought to be less morbid due to decreased uterine size, vascularity, and placental bulk [3]. In this setting, uterine artery embolization (UAE) can be used to decrease placental blood flow and bulk enough to allow for conservative management with interval hysterectomy.

Determining the clinical utility of UAE in the conservative management of MAP is challenging. Presently there are no evidence-based guidelines on its role and the little data that exists is inconclusive [2]. Information about complications of UAE is extrapolated from the nonobstetric pelvic trauma population [4]. Reported complications in the nonobstetric literature include iliac thrombosis, ischemic neuropathy, bladder gangrene, uterine necrosis, leg ischemia, and buttock necrosis [1, 5]. To our knowledge, the only two reported cases of buttock necrosis in the obstetric literature occurred in the setting of UAE for postpartum hemorrhage [6, 7]. Buttock necrosis has not yet been reported in the setting of conservative management of MAP. Herein we present a case of conservative management of placenta percreta with delayed hysterectomy and UAE complicated by maternal buttock necrosis.

## 2. Case

**Figure 1:**
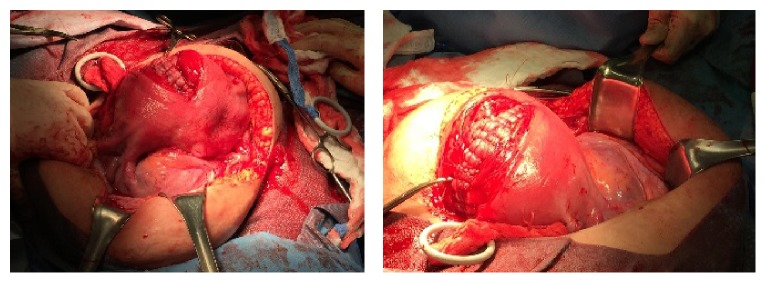
Intraoperative images after hysterotomy closure demonstrating hypervascularity of the lower uterine segment with ballooning of the right broad ligament.

A 39-year-old gravida nine para two was referred at 30 weeks of gestation for evaluation of MAP. Her history included two uncomplicated low transverse cesarean deliveries, four curettage procedures for pregnancy termination, and two spontaneous abortions. Pelvic imaging showed an anterior placenta with obliteration of the uterine interface, presence of intraplacental vascular lacunae, and increased vascularity proximal to the bladder and loss of the myometrial margin. Findings were suspicious for placenta percreta possibly involving the urinary bladder. The patient was counseled extensively regarding the management of MAP, including conservative management and its associated risks, and comprehensive informed consent was obtained. Planned delivery at 35 weeks and 6 days of gestation occurred under general anesthesia. Immediately prior to delivery, the patient underwent uncomplicated placement of bilateral hypogastric artery balloon catheters and bilateral ureteral stents. At the time of stent placement, systematic inspection of the bladder was noted to be normal on cystoscopy. Intraoperatively, an area of tortuous blood vessels on the uterine serosa was noted extending to a ballooned right broad ligament and to the border of the bladder reflection on the left side, confirming a placenta percreta with broad ligament and possible bladder invasion (Figure 1). Given the extent of abnormal placentation, conservative management was elected. Uncomplicated cesarean delivery of a neonate weighing 3,150 grams via a fundal hysterotomy then followed. After high ligation of the umbilical cord, the placenta was left adherent to the myometrium. The hysterotomy was repaired in three layers and total estimated blood loss was 1000 cc. The patient was then transferred to the interventional radiology suite in stable condition where balloon catheters were removed followed by bilateral internal iliac embolization using a slurry of absorbable gelatin compressed sponge (gelfoam). Postembolization arteriography was performed showing stasis within the uterine arteries.

Shortly after arrival to the postanesthesia care unit the patient reported buttock numbness but was otherwise asymptomatic. The epidural catheter was removed on postoperative day one and shortly thereafter she reported a burning sensation in the right buttock. Physical exam revealed a tender 9 × 11 cm area of ecchymosis in the medial aspect of right buttock with extension to the gluteal crest. Given recent embolization, there was concern for end-artery compromise and potential soft-tissue and skin necrosis. Interventional radiology, vascular surgery, and plastic surgery teams were consulted. Computed tomography angiogram demonstrated patent internal iliac arteries with patent proximal pelvic branches. Over the following week the lesion blistered, necrosed, and unroofed to reveal healthy tissue beneath. The lesion was treated with topical silver sulfadiazine cream and pressure relief. The patient was discharged home on postoperative day seven with close outpatient follow-up.

The patient was planned for interval hysterectomy at six to eight weeks postpartum but was briefly lost to follow-up. Shortly thereafter, she presented and was admitted to the hospital when she noted umbilical cord tissue prolapsing from the vagina. She subsequently underwent hysterectomy at ten weeks postpartum [8]. At that time the buttock wound was noted to be healing well with improved epithelialization, scar contraction, and healthy granulation tissue throughout (Figure 3). She was recommended continued use of daily hydrated polymer dressings until the wound was fully closed.

## 3. Discussion

With the increasing incidence of MAP and associated complications, substantial effort has been made to determine optimal management strategies. Leaving the placenta in situ is one strategy that can be employed in the case of placenta percreta when intraoperative findings indicate a high risk of operative complications. This may allow for a decrease in placental vascularity, which can lower the risk of operative complications at the time of both delivery and delayed hysterectomy [2]. UAE is thought to facilitate placental devascularization. However, there is little published data to support UAE in the setting of conservative management of MAP.

Planned UAE is most often performed using prepared embolic agents. Agent selection depends on vessel size, desired duration of occlusion, and whether the embolized tissue is to remain viable [9]. Most organs have a duplicated vascular supply and are maintained through a network of smaller collateral vessels. The risk of organ ischemia is greater with smaller embolic agents because they can travel distally to junctions where feeding arteries and collateral vessels meet, thus occluding terminal vessels [9]. The two most common agents used for large-vessel embolization are gelfoam and metal coils [9]. Gelfoam is made from porcine adipose tissue and can be made into a slurry that expands to form a cast of the embolized vessel [9]. Coils are made from steel, are coated with a thrombogenic agent, and act by inducing clot. The major difference between the two is their permanency. While coil embolization is permanent, vessels embolized with gelfoam can begin to recanalize as early as three weeks. The major disadvantage to gelfoam, especially if pieces of raw sponge are used instead of a slurry, is the risk of nontarget embolization as the small pieces can travel both proximally and distally to occlude smaller terminal vessels [9]. Use of gelfoam embolization in the conservative management of MAP has been recently documented in the literature [4]. However, we found no reports to date of complications directly related to embolization in this patient population.

The case discussed herein was complicated by terminal vessel embolization with resultant ischemia of the skin and underlying gluteal soft-tissue. Buttock necrosis is a rare complication after UAE with limited documentation in the literature, almost exclusively in the trauma literature relating to the treatment of pelvic fracture [5]. To date, there are only two published case reports describing inadvertent gluteal vessel embolization with resultant tissue necrosis in the obstetric population [6, 7].

**Figure 2:**
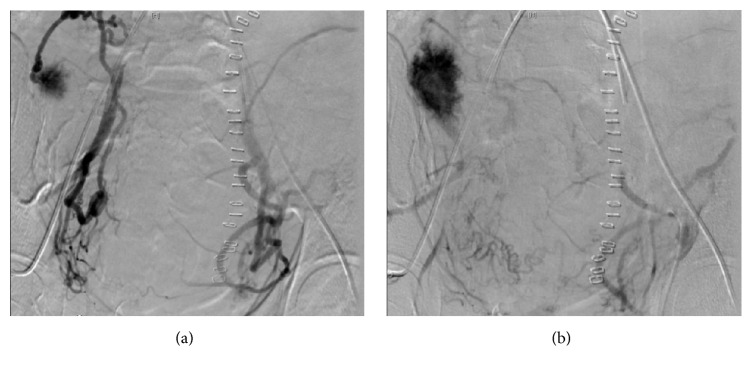
Fluoroscopy images prior to embolization of the branches of the internal iliac arteries demonstrating increased vascularity on the right branches compared to the left branches. The dark spot in (b), taken immediately following (a), indicates placental parenchyma.

**Figure 3:**
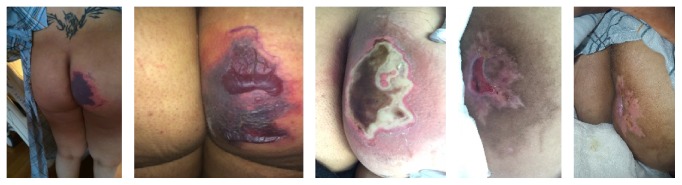
Buttock lesion images demonstrating progression and healing. From left to right: postoperative day one, day three, week four, week eight, and week ten.

Gelfoam embolization, when employed, is now routinely performed using a slurry under direct visualization with fluoroscopy. This allows for specific and real-time modification of gelfoam placement and cessation if reflux is noted [5]. Although nontarget embolization is a known possible complication of gelfoam, the incidence is unknown and likely very rare. In a case of buttock necrosis described by Al Thunyan et al., raw gelfoam pieces rather than a slurry were used. It is unclear how fluoroscopy was used to guide placement [6]. In our case, placement of the slurry was achieved under direct visualization without complication. Though a large amount of gelfoam was used, the interventional radiologists involved strongly believed the complication was not a result of nontarget embolization. The vessels intentionally embolized during UAE are known to perfuse the buttocks but the extensive collateral vasculature in the pelvis prevents buttock ischemia when those vessels are embolized, especially in the setting of increased pelvic vascularity and blood flow in pregnancy. In this case, both uterine arteries were embolized equally, as evidenced by real-time fluoroscopy. However, only the right side demonstrated ischemic changes. The placenta, which was noted on fluoroscopy to be abnormally and unequally more vascular on the right side (Figure 2), was thought to have acted as a vascular steal or “sink” which prevented surrounding tissues from providing sufficient collateral circulation to the buttocks.

While there is robust literature evaluating complications of UAE in other populations, there exists fewer data on the incidence of complications in the obstetric population. A recently published literature review aimed to compare the rate and risk factors for the development of ischemic complications after interruption of the hypogastric artery in the vascular surgery, oncology, trauma, and obstetrics and gynecology populations. In this review they reported complication rates and found that, among obstetrics and gynecology patients, ligations resulted in fewer complications when compared with embolization [10]. However, the indications for hypogastric artery interruption in the obstetrics and gynecology populations included in their review were uncontrolled obstetric hemorrhage and blood loss during nonobstetric hysterectomy. To our understanding, this is the first reported case of buttock necrosis in the setting of UAE for conservative management of placenta percreta. While UAE can potentially decrease the risk of blood loss, it is not without its own risk of complications. Given the increasing incidence of MAP and the unknown incidence of complications related to conservative management, a multicenter case series addressing potential complications is needed.
